# Congenital Imperforation of the Urethra the Whole Depth of the Glands

**Published:** 1847-11

**Authors:** E. Whitehead

**Affiliations:** Surgeon, Dakinfield


*Congenital Imperforation of the Urethra the whole depth of the Glands. By* E. Whitehead, *Surgeon, Dakinfield.*—Mrs. B. gave birth to a male child on Christmas eve, 1846. I called the following day, but no remark was made. Being a distance of a mile and a half, I omitted visiting on the second day, but intended doing so on the third. However, early on the morning of my intended call I was hastily summoned. On my arrival, I found the infant screaming vehemently, and apparently in great pain. Upon inquiry, I was informed its bowels had been moved repeatedly; but the nurse could not say it had ever passed any urine. Upon examination this was verified, the glans penis being imperforate; bladder considerably distended. Not having my pocket-case with me, I ordered the little sufferer to be brought to my house, where, assisted by my friend Mr. Hunt, surgeon, of Ashton-under-Line, an opening was made with a lancet in the place where the orifice of the urethra is usually situated, and an attempt made to pass a probe; the lancet and probe were again used, but without success.

The incision was then carried through the inferior length of the glans, upon which urine escaped freely. Not the slightest vestige of an opening could be found in the glans other than the one made by the operation, which was allowed to heal. The artificial outlet now supplies the place of the natural one.—*Ibid.*

